# Great Dental Patent Case

**Published:** 1855-07

**Authors:** J. Allen

**Affiliations:** 30 Bond Street, New York


### Argument of Stanley Matthews, Esq., for Defence.

I shall not attempt to disguise the fact, that I approach this case with some solicitude. It is an important one to the plaintiff, and not so insignificant to the defendant, as the attempt made by the venerable gentleman who has just closed, to show up, the private and professional character of Dr. Hunter.

Personally, I must premise, I am a novice in practice in the U. S. Court, appearing now for the first time before these honorable Judges, in a litigated case. I appear for the first time in my life in a patent case. I present myself single and alone, against an array of talent from the Bar, the solidity and strength of which cannot be excelled; but with these great disadvantages, I rely confidently upon the justice of my cause.

On account of my weakness in this contest, like David with the Philistines, I have armed myself only with a sling and a few pebbles of truth, with which I expect to penetrate the armour of my adversaries. There are three persons of the opposite counsel. My friend who is to close, is the Hamlet of the play. One of the venerable gentlemen is the Polonius, to nod assent at intervals, and ejaculate on suitable occasions, "very like a whale." But this issue is not to be tried on professional opinions; the contest before you is on scientific principles ; after all there is but one true test, and that is common sense—that probes every theory, dissects every opinion, and tries every conjecture of science—whilst they weigh us down with abundance of witnesses, we, with a few grains of common sense, hope to precipitate the turbulent waters.

As to the contest of the two men, the two professional men, I do not approach it, but withdraw from it. I have in this case no other feeling than the usual sympathy of counsel and client.

As to the crimination and abuse alleged,—Dr. H. has been in no combination, he was in his Laboratory, and had not time to spare from his experiments to travel around from Association to Association to exhibit, unformed, unfinished, and impractical specimens of work—to blow a trumpet of what he was to do—he did not attempt to forestall, but to present results.

At one fell swoop, gentlemen who left their business to testify under oath, not to vindicate Dr. H. but to tell the truth, have been indiscriminately charged as perjurers and scoundrels.

I was astonished that a man whose brow was frosted with age, would take occasion to involve all in one common conspiracy of crime.

But this is not the case before you, you have had the witnesses before you, heard their cross-examination and replies—you are the Judges.—From the course taken by the venerable gentlemen as to the pamphlet. I did not know but what he was going to include, you, gentlemen of the jury, in the conspiracy he alleges have had such designs on his client. I shall not attempt the vindication of the witnesses or my client. The hot blood of the old man must be suffered to cool—the blood of Douglas seems to be with him yet. I shall indulge in no such personal tirades, even if my taste might lead me so to do; the necessities of my case do not require it. I object to the Patent as being void on its face for ambiguity; in this, it is impossible to say, from the Patent, whether the Patentee claims a new mode of setting teeth upon metalic plates, with continuous gums, by means of the *particular* compound, which he also claims, or by means of *every* fusible silicious cement, however made or composed, which may be used to produce the result. I expected to have been enlightened on this point by the counsel who opened the argument for the plaintiff.

The patent covers three claims.

1. The covering of asbestos and plaster, on which point there is no controversy between us.

2. The cement, consisting of any known flux, combined with silex, wedgewood and asbestos.

3. The mode of its application, so as to produce certain new and useful results. Is it an infringement if Hunter's plan produces the same results?

Results are not the subject matter of patents, means, in certain combinations, may be, although they be as old as Adam's time. The patent is for his means, not for his results. The opposite counsel says it is, if so, the patent in that is void. You can't tell from reading the patent, what *certainly* and *precisely* the claim is for.

It is left often to the Judge to pronounce patents void for this uncertainty. (See Vol. 6, English Law and Equity Reports, p. 272, also p. 409,) where on the ambiguity of a single word, a qualifying adverb, the patent was declared void. In all my study of this case the ambiguity continues.

Even if by a liberality of construction, your honors do not make such ruling in this case, yet they must be held bound to substantiate all three of their claims; covering, cement, and application of continuous gum to plate, which it is claimed, produce new and useful results.

As to the covering of asbestos, I believe it is not alleged that we stole that. As to the *cement*,—the claim is for *every* cement which can be formed by the combination of *any* known flux with silex, wedgewood and asbestos. This means just what it says—any flux then known to the public or to science.

It has been tried to limit the matter to such as tooth makers. I controvert it—it cannot mean such fluxes only as were known to tooth manufacturers, or block workmen, or dentists generally. 1. Because the language is explicitly otherwise. 2. Because feldspar and glass were the only fluxes known to them in the practice of their art. The patent was addressed not to the outside knowledge that men of science may attain; but to ordinary and average knowledge how would they construe it? Dr. Wildman, who used borax, kept his receipts secret; they were known to but few, and were not published until 1853, after the date of the patent. 3. Because the ingredients in A.'s compound were known in other arts, as the manufactory of pottery, porcelain and glass; also, the enameling of pots, &c. 4. The compound and its uses were perfectly new; on the hypothesis of the patent being for a new discovery to dentists, tooth manufacturers and block workmen; and no previous knowledge of the preparation of similar compounds, with fluxes for similar purposes can be supposed. He claims it is new; a man can't patent old means to produce new results—he claims it is new, and known only to him.

The claim set up, is for every cement which may be formed by the combination of any known flux with silex, wedgewood and asbestos, without reference to the proportions of the ingredients, or the manner of compounding. It is not merely for the formula which the patentee says he prefers, but for all, universally, however compounded.

It is not however for a cement, formed by the combination of a flux with but one or a portion of the other ingredients, or with the whole of them and some additional ingredient, which has a specific effect of its own, in determining the character of the compound. Neither doos it include the same material with a flux unknown at the date of the patent. What then does it cover?

The claim of the patentee covers the following particulars:

1. Its inter-mixture or underlaying with gold and platinum scraps.

2. Its application, so as to produce the desired result, upon plates constructed and teeth arranged thereon in the usual way, and of plates constructed of the usual materials.

3. The arrangement of the cement upon and around the teeth, so as to form an artificial, continuous gum, adhering to and uniting the teeth to each other and the plates on which they are set.

4. The covering of the teeth and plates by means of a composition, so as to maintain them in their position, while the cement is being fused.

5. The fusion of the cement in the furnace in *one* heat, and the application of the gum color also in *one* heat.

6. The dispensing, by these means, with metallic back plates, solder and blow-pipe.

7. And, also, that back plates may be attached, in the usual manner, if desired.

These seven points are embraced in the patent—some are methods and some are results—they constitute the essence of the claim.

This construction of the patent being established, the question then comes up, can a person of ordinary skill in the dental art, by following the directions of the patent, without invention or experiment, successfully produce the results? (See Curtis, p. 162. Webs. 37, 53, 170, 218.)

The patent is addressed to persons engaged in the business of setting mineral teeth upon metallic plates, not to tooth manufacturers or block-workmen; and not to those of the greatest skill among them, but to those of the average and ordinary competency. (See Caveat.)

It is addressed to those of ordinary skill in the dental art, who are without other information except that obtained in their ordinary practice. This question arises in this way. The Statute makes the patent grant on consideration, not of $30 paid in, but for such a disclosure of the discovery, that if the inventor dies within its term, or afterwards, the knowledge of the mode may not die with him, but the public may derive the advantage and readily produce the proceesses the patent describes. The statute requires then the statements to be full, clear and exact in their terms, enabling persons skilled in the appertaining art so to produce the new and useful results. (See Curtis on Patents, p. 156 to p. 165.) We contend then that the language is not full, clear and specific; we say that a person but ordinarily skilled, cannot possibly produce the results claimed, by adhering to the directions given and the processes described.

1. He could not form the *compound,* if he followed the formula prefered by the patentee; he could not make it without fritting the borax, a process not laid down.

In the case, King vs. Arkwright, the most valuable of inventions was declared void for ambiguity.—Also note page 162, Neilson vs. Howard.—Also Sections 157, 158, Curtis.

You have heard of borax and the water of crystalization, and can see the dilemma in which he is placed who proceeds to do work under the patent. Though but one kind, yet borax is found of four various conditions—which shall he take? Shall he be left to guess and to experiment? Not a witness appears, but testifies that the water must be driven off—they say any man of sense would know it must be expelled—he would immediately know that the specifications were false—the business of the patentee is to tell the facts in his language to the public—a patent of a French chemist, for sugar refining, was blown sky-high for a no greater defect than in this, of not directing to *frit.* (See Curtis on Patents, Sec. 162.)

Allen afterwards gives instructions that it must be fritted. He spoke of the witness who appeared for A., who although he said he had been a dentist for 14 years, he did not know what a "muffle" was. For a compact statement of the law of patents, See 15 Howard, p. 119. The man of ordinary skill then, could not know what kind of plates were necessary. The patent tells him to construct his plates in the usual way; the usual way is to take a piece of silver or gold of 16 to 20 carats fineness, and shape them to the mouth—this would not answer.

Suppose he should know, that such a compound would not fuse without melting such plates. He is bound to follow the patent and it only proves that he would know the patent was worthless.

It is of the essence of the patent that this work is susceptible of being successfully executed upon "plates constructed in the usual way" if it cannot, the patent fails. But he could know no such thing without previous experiment.

Platina plates must be constructed in some other than the "usual way," to answer the purpose, they must be banded, rimmed, &c. He could not make the work by one fusion of the gum body. He could not dispense with metallic backplates, solder and blow pipe. If he wished to attach backplates, he could not do it in the ordinary way, because the heat would destroy the solder, and no other way is pointed out. (See Webs. 80—Curtis p. 193, 291, 432–4, Barr & Ald. 541.)

Again I say, what borax would he use? He has only been accustomed to and can only purchase, the borax of commerce. Except he be learned as a chemist, he mixes and puts on the gum body and puts the specimen in the furnace—the result would be like one of those presented here, blown up into a mass, like parched pop corn. Men in good faith have done this and found that they were wrong, until they had experimented—but the patent says you must do it without experimenting; he is told he may use any known flux, he is at a loss which to choose, to know what will do—the oxide of gold, of tin. nitre, sal ammoniac, black bottle glass are fluxes, but these can't make it, most assuredly if he deserts borax, if he takes it, no proportions are provided to mix the compound. Suppose he bought the compound of Dr. Allen, made after the private formula," being a good Dentist, a good block maker, what does he do? He takes, as was his practice, silver or ordinary gold plate—not the alloy described by Van Emon of from six to sixteen carats, but at least a decent article from sixteen to twenty carats fine—he swedges it up, arranges the teeth thereon, with backstraps and solder, takes a gum, arranges it, puts on the asbestos, puts it into the furnace, it fuses, he pulls it out, he beholds with amazement, that the plate has dissolved, silver will not do, an alloy of gold and silver won't do. What shall he do? He reads from an old author, or gets verbal instruction from Dr. Allen, and learns that platina *must* be used, that he finds won't melt. His patient is waiting, a single plate is too weak, he has then to invent some plan, proceed to rim, wire, band and double, applying Cleveland patent air chamber. That is the experience that even a skilled person would fairly and honestly have to pursue. Persons, it is true, have said, that they have made the work—were they persons of mere ordinary skill? It is very easy to do it when you know how, but that you don't know fully from the patent. Many could do work after Dr. Allen had been around lecturing.

As to the witness Chapman, if we had had notice to have cross examined him, we should have found, as we have in other cases done, he succeeded, but not by following the specifications—no such work can be made by them.

Dr. M. W. S. Kendall says, "Common sense would teach him." Common sense? I think I have common sense, I know you have, but how could common sense tell you or me, when the proportions of an experiment had never been tested by us, what degree of heat for fusion was necessary?

All the D. D. S.'s in the country may unite in the like affirmation, but yet a queer conception of its farcial absurdity will be awakened in unprofessional minds.

I don't deny that with very small leverage two teeth may be so attached as to resist the force of human fingers, but make an arch, a complete half denture, without back plates and apply it practically and it will not last three months. When and where did Dr. Allen make such work? He may have done such in his laboratory for experiment, but when in the mouth, it has failed; it has not been done. That brilliant luminary has not been so refulgent in his full day splendor as in the blaze of his office lamp and blowpipe. Erudite, learned, laborious, as he is, he has not done it; yet in the articles, the printed publications which have been read and which I now read, he says he has. Now I am satisfied that by experiment, by actual test the claims of Dr. Allen have not been verified. Nearly all the witnesses say that the work will not do, without back plates, that they are not worth anything.

Dr. James Taylor says that for a few teeth they *may* be dispensed with. But that is what Dr. Allen peculiarly claims in his invention, that he has discovered a plan in which back plates are no longer needed—that was the great desideratum—so he says in his article—so he sets out in his Patent. Now he has not proved that claim or produced evidence thereof—if serviceable teeth are found it must be with back plates on. If back plates are put on, how, under the want of directions in the patent, shall they be put on? Shall they be put on "in the usual way?" If so, that is by solder.

Having tried it, common sense would tell it would do so again. It was contended by Allen himself, before this suit was brought, that *gold* would answer. Had he no common sense? He read from Allen "The ordinary gold plate can be used but not with perfect safety, probably &c."

"I use gold with no alloy, but I use platina, which I think better" he put on none, that stood, but platina. Is there then no invention, experiment, to ascertain that platina is necessary? When in burning, checks are found, how does the new operator know that he must fill up the checks or flaws with the same material? How does he know that he must make it a little more fusible—these instructions are laid down in the "private formula" not in the specifications and this is the way, the proof shows, that the work has been done.

What the purchaser of a patent wants is plain unerring instruction for the certain and profitable use of the invention. He is told to get up his plate in the usual way. He puts on his body, fuses the compound, puts on the gum color; it appears, say, good; it is put in the mouth, but how long does it stand? It soon crumbles and gives way.

But we are told that we have the certificate of Prof. Thomas Wood that in his hands, in the presence of the whole class, two teeth on a plate, as an experiment, could not be broken off, that the force necessary to force the gum, would be that to crack a hickory nut, as to this matter, I do not wish to call hard names; but to me this seems a monstrous professional extravagance.

What solder shall he take?

He takes common solder; he fuses his solder, but lo! his work is gone with the solder, his back plates are there but no attachment. Thus by successive experiments, at perilous cost, by five distinct inventions of the operator, the work may be done, and thats the way the proof shows it has been done.

Then as to infringement—(Webster, 1852)—there is no proof of any such work done by the defendant, after the date of the patent. (Mrs. Hannah Steven's, April, 1852.)

The Counsel on the other side expected us to come down, pay 6 cts. damages, pay the costs and back out—if there be any infringement between us, it is he on us, not we on him.—You have not proved a case on us of using his compound. There is some law on this point. (25 Vol. English Law and Equity Reports, p. 535.) There is no evidence of our using his mode; they must show we made his, using substantially the same compound. Where is the set of teeth of Dr. H. made in the same way? There are none presented. They say that his compound as published in the "News Letter," is the same as A.'s. We deny that; it is a different thing.

Hunter's compound differs substantially from Allen's:

| ALLEN'S. | | HUNTER'S. | |
|---|---|---|---|
| Silex, | 2 oz. | 1. Granulated body | |
| Flint glass, | 2 oz. | Spar, | 3 oz. |
| Borax, | 1 oz. | Silex, | 1½ oz. |
| Wedgewood, | 1½ oz. | Kaolin, | ½ oz. |
| Asbestos, | ¼ oz. | 2. Flux. | |
| Feldspar, | ¼ oz. | Silex, | 8 oz. |
| Kaolin, | ⅛ oz. | Calcined borax, | 4 oz. |
| | | Caustic potasa, | 1 oz. |
| | 7⅛ oz. | Take Flux, | 1 oz. |
| | | Asbestos, | 2 oz. |
| | | Gran. body, | 1½ oz. |
| | | | 4½ oz. |

The differences between Allen's and Hunter's are :

1. Allen's is a simple compound; Hunter's is a compound of compounds.

2. The proportions are different.

3. The fluxes are different.

4. The mechanical structure is different; the one is a coarse powder mixed with a fine one, the other is an impalpable powder.

5. The principle of the compound is different: one is a glass, the other a porcelain ; one is homogenous, the other not ; one is thoroughly vitrified in all its particles; in the other, the flux vitrifies and coats the surface of the grains of the infusible body, supporting each other, and maintaining the form of the mass.

6. The ingredients are different.

7. The results are different, one contracts in fire, the other does not.

Much has been said of the sameness of compounds in their "ultimate analysis." They may lecture all day on the identity of an oak and a pine board, but they can't prove that a bee hive is a board fence.

One of the witnesses being questioned on this point, says, that the ultimate analysis of the world shows all its materials to be the same —it all came out of chaos, but that has no application to a case like this. The chemical analysis of two human bodies are the same, but their indentity is different. Dr. Allen don't patent chemical results. He takes certain things. Hunter certain other things. Dr. H. don't use asbestos at all, and uses what Dr. A. does not until after H. made his formula known. There is no caustic potash in the patent; there is in the "private formulas, subsequently accompanying the same.

If you remember, I put it to Dr. Slack, if the effect of similar things uncompounded was that when compounded. I asked if there be potash in glass, will glass laid on the surface of the flesh burn; will it not in its natural state have that effect upon the sensitive human body. Water consists of hydrogen and nitrogen, but neither of them is water. Send a stroke of electricity however through them and the result is water. Take one grain of calomel and take another such a grain and mix with the sugar of milk and one-tenth of the latter is more powerful, medicinally, than the whole of the former. When we come to the ultimate analysis, we go beyond our comprehension; we seek to realize principles too deep for us to fathom.

It was a question to the distinguished philosopher, Sir Humphrey Davy, why a tub of water with a fish in it weighed no more than the same without the fish. He gave the matter up, but was more astonished when he found that the fact was not as predicated.

Dr. Slack's opinion as a chemist is entitled to great respect, he is entitled to much respect as a man; he is an old citizen among us. He in early days was President of Cincinnati College and Professor of Chemistry in that Institution. Perhaps an historic incident giving an example of the effect of experiments, as practised under the eye of the venerable Dr. Slack himself, as once told with eloquent power by the late Benjamin Drake, may not be unprofitably related here. The old College, as many of us recollect, had a high range of steps from Walnut street, while the main hall on the first floor was accessible more easily from the rear, where the pioneer grave yard had been allowed to go partially to waste, and the cows if chance occurred, would avail themselves of a brouse on the grass that sprung up around the borders of the sunken graves. One morning, by a mischievous arrangement of some of the boys, a cow was found in the Chapel at the time when College prayers were to be said. The tutors were unable to remove the *muly*-ish animal, (in more ways than one.) In came the President, Dr. Slack; he surveyed the locality and all the parties. He ordered the cow to be ejected *vi et armis*." Two boys were directed to take hold of the hornless head and one of the tail. The two pulled and the one twisted and all hallooed, the cow and parties progressed to the head of the stairs at the front entrance; there the cow chose to stop and ruminate; the two pulled and the third twisted, but the cow plunged and bellowed but would not descend. Here was a wearisome crisis—the Committee warm and weary resigned.—The President in his gown, with caton in hand, presided and directed, but that direction the cow would not take. Expedients were proposed. Some energetic "Young America" student of Dr. Slack's Chemical class, proposed that if the Galvanic Battery be applied, the cow would be put to her trump. No sooner suggested, than assented to by the President. The Battery was brought. The union of metal and acid took place, and when the electric current was applied at that point of the body where the tail is joined to the same, there was a *result*. Instantaneously, cow, Committee, President and students were at the foot of the steps, near the corner of Fourth and Walnut Streets. Reduce that gentlemen, to its ultimate analysis, if you please.

Again, gentlemen, this process patented by Allen, was made known 30 years ago by Delabarre. Take either Fitch's or Wildman's translation, and you have a better description of the principles of continuous gum work than in the specification of Dr. A.; the one is meagre and insufficient, while the other is full and scientific. Take Delabarre—what if he never did practice it—the law does not require it. If he describes it, so as it can properly be made, Allen made a great mistake in saying he was the original inventor. They say for A., he had not time to read Delabarre in French, or Fitch in English.

We have witnesses who say that they can't make work after Delabarre. We have witnesses with ordinary skill, who say they can't make work after Dr. A.'s formula. They would have our Kendall go to France to get French paste for his experiment of Delabarre, while their Kendall can take pottery porcelain, not fit to make flower pots, from that old dilapidated institution of 20 years ago, the Brighton Pottery. Then as to the prior knowledge by others.— It is proved that Dr. H. showed samples at Dr. Brown's in 1850, and exhibited the same at the World's Fair.

Take the case of Dr. Main. Take the case of the set stigmatized as being set on mud; the case of S. D. Wilson, made by Beers & Morgan; these were all known prior to the date of the alleged discovery by Dr. A.

But what is more important to us then, is the prior invention by Dr. Hunter. Review the history of his experiments and his results. Trace him as far back as 1844, with Dr. Cook striving to secure a non-contracting body for tooth work, and for a gum to flow on to a metallic base; witness, in 1846, the results spoken of by the so styled "pettifogging" Leslie; in 1847, the first weeks labor in the Laboratory, of the boy Kendall; the observance of work and experiments of Hunter in the same year, testified to by Dr. Crane, in 1848, by Dr. John Locke and John G. and by E. P. Jones. Bring to mind that long and faithful course of experiments and the perfect and finished results. They are in bodily form now—they have been in continued use since. Are these Dr. Hunter's manufacture of teeth in the mouth of Dr. Taliaferro? Did H. make the continuous gum before the date of this patent?

There was the formula sold to Jones, W. & Co., Philadelphia, in 1850; the London Fair work in 1851, and the practical work in Mrs. Guilford's mouth, still so strong and beautiful.

They appointed a Committee to examine one of the early sets made by Dr. H., and they do not see fit to ask for a report. It is a rule of law, that those who have the first start in the race of diligence should have the advantage. If Dr. A. had 20 years start in experiment, it seems that he needed it. Hunter has the first results; there was no propriety in this exclusion, because one is a fogy man, and the other an active minded "boy."

They say that Fitch unites in degrading Delabarre, they would make out that the French author was lying to the French public, and that Fitch knowing his deceit still became his translator and recommender of the process to the American profession.

Delabarre has been quoted by all prominent authors, and Fitch says that Delabarre's plan was a great improvement, he does not convey the idea in his book that the work was impracticable—Delabarre says his work is not a new theory.

Take that specimen from the jaws of Aaron Burr fixed by fusible cement, does that look like impractical work?

I take up this book written by Dr. Jobsen, who has been a witness before you. This distinguished Litteratteur and Professional man and author, this for a long time an observer of the best specimens, writes as follows: "All mineral teeth are a deceit, they are not durable, they are brittle—easily broken by contraction and seriously injured by friction." Yet he lauds the Allen work. This was his opinion in '34 and '35. He must have changed it since. (Jobson—I have not changed.) If you have not changed, it is time that you had. He says A.'s is splendid work, yet when he is shown specimens of identical work in appearance, when he is shown the specimens on exhibition at the World's Fair, London, (through which Palace of Industry he walked without giving it a passing glance,) he carps and criticises, and says it does not compare with the set seen with A.'s agent in London, nor with specimens of block work made by the best London Dentists.

(Mr. Stanbery—I contend that no specimen of Continuous gum work by Dr. H. was ever exhibited at that World's Fair.) Then you take our witnesses to be willfully corrupt and shameless liars.

Delabarre's work we know what it is, we have tried it on—it is at least as good as Dr. A.'s.

You will remember the specimen made by Dr. Allen and exhibited by Dr. Curtis. That was a rare accomplishment for a man that had been so long experimenting—a gum that could be scratched from the plate by the thumb nail, which was actually done in your presence and that of the Court.

Then the specimens of Mrs. Aldrich were also easily scratched. B. D. Wheeler the brother-in-law of Allen testified that the teeth of Mrs. A. were set on gold plate. In 1849 Dr. A. was full with his result. He showed to Leslie his enamel specimen, when nearly a year previous, Dr. Hunter had placed good servicable teeth in the mouth of Dr. Taliaferro.

The authour of that brass back plate work and the brass pin was bragging what a great man he was going to be when his design was completed, while in 1849 Dr. Hunter was making an alternate set for Dr. Taliaferro, and in 1850, sold identical formula to Messrs. Jones, White & Co., and published the same in the Dental News Letter.

And here as to fraud alleged, it does me good to observe the ingenious artifice. It was by the other side attempted to be conveyed that the sale to Jones, White & Co. by H., was in 1854, when by the letter written and recognized it was ascertained to be as early as the summer of 1850, eighteen months before A. got a patent.

Did Dr. H. send continuous gum work to the World's Fair? Did he purchase the teeth and platina from Brown for that purpose? Did he show the finished specimens to Crane, Leslie, Toland, Brown and others? Could there be a mistake? Was it not described to Taylor? Did not Toland tell the same to Allen? Did not Brown also communicate to Allen what success Hunter then had?

A. knew of it prior to 1851, for Brown in answer to his queries as to it being only block work, replied that it was continuous gum work. In the succeeding fall A. showed a piece of enamel as the result he had attained. This was the same as Hatches' teeth—it was not continuous gum work. Hunter however went right on. It is immaterial whether his specimen was in London or not; it matters not whether it was sunk in the middle of the sea, that would not entitle Allen to the patent. Suppose it had been stolen from H. and given to Allen, would that give the latter a right to claim its invention?

If H. substantiated its existence as his work, it is enough.

But that was there. We have the Commissioner's Certificate. Kinsly saw it before and while at the Crystal Palace. Where is the specimen of A.'s work made before the patent?

In April 29, 1851, by the Caveat (which see) he gave notice to the world that he was still experimenting—what he had attained then, you find in that Caveat—you have the formula of material, but no proportions.

We thus let you into a secret. A. became acquainted with Steemer, the tortuosities on both sides of this matter, I do not propose to follow, but A. bought of S. his receipt in May '51. Suppose it was good for nothing, it can make a gum; does he deny it? compare it with the Caveat—it shows the use of a portion of Steemer's formula, that A. was still in the path of experiment. It shows that A. as yet had discovered no cement. He got his patent out in Dec. and of course had just perfected his experiments—if his work had been in use two years previous to patent, on that account the patent would be outlawed. After H.'s publication, every time A. got a new idea he wrote new directions in his private formula.

He once said to the profession here, that he had got out a Caveat but although filed in the Patent Office, *there*, he said, it might be be forever, he intended to lecture on, and thoroughly make known his process to the public. He has concealed a number of points vital to success and important to be known.

Arkwright from patriotic motives, fearing the French might pirate his invention concealed many important points, and in spite of his name and character, of his patriotic intent, the patent was taken from him.

I have striven in my feeble way with candor, frankly and honestly to try this case on its merits. This mode of adjustment instead of being reluctantly brought on by Dr. H. was sought by him, sought in behalf of the profession to understan l his right.

His design and desire has been to let all come out, to let the whole be told and all be heard before an impartial Court and an unprejudiced Jury. If Hunter has infringed give the verdict against him. If Dr. Allen is not entitled to the patent, let no prejudice or sympathy have aught to do with the just determination of the issue. As to the aspersions cast upon the defence they are not noticed. As to Dr. Hunter, I feel proud that I have such a man for a client. That is all the vindication that he needs.

Mr. Matthews concluded at 5 P. M., Friday evening, when the Court adjourned.

### Closing Argument for the Plaintiff. Speech of Hen. Stanbery, Esq. Saturday, 9 A. M.

The position of Judge Coffin as original Counsel for Dr. Allen, entitled him to the closing argument, but he waived his privilege to his associate, Mr. Stanbery, who proceeded as follows :

*May it please the Court and Gentlemen of the Jury:*—

I can't disguise the fact that I feel great anxiety and more than ordinary responsibility in approaching the consideration of this case. My client is entitled to my best exertions, and I know I can't come up to his best expectations or to the necessities of the case.

The advocate who has to defend Dr. A. in a controversy managed on the other side by one who challenges our respect, requires an ability and a preparation that I was not able to make. Being but a short while since called from professional engagements to unite with Counsel in this case, my time has been so short that I feel my want of preparation.

I feel as if I ought to have had it, to have made clear the justice of my client's claim, and to have defended him from the attacks made upon him.

You have attended so far with patience the argument of my learned friend of the defence, who has conducted his case with so much ability. You must not prejudge the case until all is heard.

It has been for a great many years the business of my life to try cases, and in this one I am not lost to a proper sympathy for my client.

I confess myself not made of refractory and infusible materials; that while I know there is a right on one side there has been very great wrong on the other. My client has an adversary that no one can meet without dread ; a man of untiring energy—and I wish I could stop here—unscrupulous and very vindictive. He has pursued an unoffending man for years, to hunt him down ; he has been challenged to this suit ; been badgered by means not only unscrupulous but very vindictive, and more, not based in truth. My client deserves encouragement and thanks, while the other side has given him curses and maledictions. He has wrought diligently, for years, in pursuing his experiments and improvements and now, when he was about enjoying the fruition of his labors, the defendant stepped in to take away his professional honors and distinction. This he has done in various ways. By alleging that the invention is not a new one ; that the idea was that of Delabarre, Dodge, Steemer, Levitt, and last of all himself. I say Hunter never dreamed of it. If there is any good in ours it is A's; if there is any good in H's, which I do not deny, it is not his invention, it is A's.

Now, gentlemen, one word or more with our friend Matthews, who has arrayed the parties here in a skillful manner. He characterizes himself as a David, a shepherd boy prepared with stone and sling only, to contend with a giant armed from head to foot. I have no objection to his name being called David. It is, however, not every one who throws stones that is a David. I see in him the evidence of success ; I am pleased to say this, but no ability in a bad cause will atone for such a sacrifice, and while one stone may be successful in a good cause, a hetacomb in a bad may not avail. He saw fit to speak of me as the Hamlet of the play. He was not a Dane ; but in one sense he might not be unlike the historic character to which he had alluded. Hamlet acted a part in developing a plot—in revealing a conspiracy. This case is not one of taking the life of my client, but is a conspiracy against his good fame and to deprive him of reaping the rewards abundantly his due.

The case deals in a new subject different from our usual, ordinary experience, requiring us to be students to learn. The patent is for an improvement in the Dental art—not for complicated machinery; but for what was invented after long experimenting to produce. It is an American, a Cincinnati invention. It is said, the invention of *two* Cincinnatians. That two Cincinnati dentists, at the same time, should have hit upon exactly the same experiments, used exactly the same elements, made exactly the same compound, produced exactly the same results, which, when shown, the difference between them can't be realized, and that, in the same town, seems incredible. Gentlemen, it never was so. One or the other has taken it from his fellow. Which ever be the inventor we feel proud to say he is an American. In the United States is the place to look for experiments in the Dental art.

Dr. Jobson, from England, an artist and an adept in science, has witnessed with astonishment but delight the present elevated standard of the American profession in Dental science. The reason is, it is the only one in which we have had a fair start with older and other countries. They, for centuries, before this country was settled, had their operators trained. This, so recently a science, was, but a short while since, a common art practiced in the rudest manner. In my own remembrance all the practical service was performed by traveling pedlars of the art, whose highest attainments were plugging and filling or pulling. Neither is the skill and progress in this similar to the cognate arts. Go to all European cities, to all of the old world, at the head of the Dental profession stand Americans.

The last result attained by us in the march of improvement is as near nature as possible. Now, that this great improvement was made by Allen or Hunter none can deny. It has not yet been four years since it was patented, and is getting into general use, although the Defendant and those colleagued with him have attempted to cry it down. It has already been recognized in New York where all great enterprises begin and where all go to make a start. It has met with favor in European cities, and the only wonder is that it has grown so fast under the discouragements it has met. It has met opposition from certain professional men accustomed to the old mode, who plead the old maxims "Everything new is not true; everything true is not new."

Take the discovery of the power of steam. What good did it do Fulton? Look at Watts and the steam engine: it met with impediments; had opposition at first, but eventually it filled the world with its triumphs. No wonder that in this new line of improvement, those who tried it were delighted with it. Let those carp at and decry it who will, it speaks for itself. One or other of these parties arrived at their result first. If H., never, for a thousand years, could apology be sufficient for the claim set up. If A., can H. ever make reparation for the wrong done?

The discovery has been made; a certain siliceous compound fused, at a certain heat, on a plate, causing an union of the teeth to the plate, making solid work, so perfect and entire, that besides its beauty, it is durable; resisting the action of the natural secretions of the mouth; having no angles or cracks, but fashioned after the natural conformation of the mouth.

Here are the specimens, gentlemen, which you have seen and admired; the most splendid specimens of the Dental art any one ever saw; every particle of the surface of the compound touching and uniting the teeth to the plate.

1. There is the compound;
2. The admixture of plaster and asbestos, to hold the teeth in their plate;
3. The application to the plate, by fusion, uniting the mineral and metal.

The plaster and asbestos are as necessary in the construction as the scaffolding to a building.

As to the ambiguity of the language of the patent, that subject is not for your consideration, but for the Court. We claim these three as new things—as a new method. We have patented the compound, the mode, and the application. If not, this would be be mere leather and prunella.

As the eminent Judge is thoroughly experienced in the consideration of patent controversies, I most readily leave with him the subject of "ambiguity."

How different the grounds of the Defense. They attack our specifications, granting that we did invent, claiming that the patent is void for ambiguity, that our language is not clear. Again, they say we never invented what we claim to to have invented; that the process is not new; that it was known and practiced by Delabarre, Deserabode; that Fitch's description made it known and read of all professional men. They say that work was made in Buffalo. No, gentlemen; no such work as ours has been made before.

They say we got it from Levett, that we invented nothing; again they say we bought it of Steemer, a German operator in this city in May, 1851. Last of all, they say Hunter had previously made the work; there has been a good deal of change in ground assumed; the answers are remarkably indefinite. If the counsel opposite is satisfied to enter into this controversy with a sling, and but one stone; let him fill his pockets with such like.

If Hunter be the inventor, why not rest on that? Why not meet the question there? Why go back to show what had been done by others? Why not be contented to show they invented it? When he announced his invention he did not give the credit to others. This is blowing hot and cold.

How happens it that Hunter went around charging $100 for instruction, if his was not a new invention? He says, his is not like ours. His is like ours since 1852, when he obtained a copy of our patent. We have shown you, that he never developed a process like ours until he saw ours. Hunter never dreamed of this invention. This conclusion is drawn from a reliable source—from Hunter himself. They say that H's process is not ours. Here are our specimens; side by side are Hunter's. Who can see, at least, any outward difference.

We published first, he afterwards. He sent and got a copy of our specifications. The specimens are alike as two peas. On exhibition is there any hidden, radical, substantial difference between them. To resolve any doubts we have called only those who can determine this question. Dentists can't. Those we have relied upon are chemists who analyze and resolve; who experiment to see if there be any apparent difference, or if they be identical. It is their business to follow out such analyses.

The Defense had Dr. Locke, Sr., for a witness; but they never asked him to take the compounds of Hunter and Allen, and analyze them to see if, by ultimate analysis, they were substantially or nominally identical. I regretted to see Mr. Matthews affect to ridicule science. The chemist is the person to make those scientific examinations which aid us, unskilled men, on these practical points. We have for this purpose presented you "Old Fogy" and "Young America;" they are both together here, Dr. Slack and J. M. Locke. The former says the two compounds, on analysis, prove to be as nearly identical as the combination of 3-|-1=4 and 2-|-2=4. What difference is there in the materials. Read Allen and read Hunter; certain names, it is true, are different, but analyze them and they are found to be the same. It is truly alleged that analyses prove that there is a common origin to all things. Silex is a simple article, not a compound substance. In Hunter's formula there is an unnecessary attempt to confound, to hide known things. Caustic potassa is in three of our ingredients. It is not the caustic, but the potash—potash is the thing. Potash we have in the spar, in kaolin, in white glass, of which it is an important ingredient. Let potash go into the furnace as it will, it does its perfect work under heat as well when in glass as if put in as potash. What is his so called gum body? He calls it a paste; a flux; makes a new nomenclature. He has got every identical thing that we have; he brings them all into the base: he has, at least, six out of seven of our ingredients. The virtue is in the ingredients, not in the particular combination of the particles in certain localities of the ingredients; they do not work as ours do, he has got, at last the things we have. These chemists say that all the physical properties of the A. and H. compounds are substantially identical; they fuse at the same temperature; they fuse at a certain heat. At the same precise heat, both fuse; they expand and contract alike. Prof. Locke says that the difference is hardly appreciable; that of Hunter's minutely less, but nearly identical, so that, if another experiment had been made the contraction of Allen's might have been less and that of Dr. Hunter more. Then their identity in melting and cooling; they contract alike; are equally hard, equally infrangible, equally infusible by the acids of the mouth. They are the same. This is a question of law also; for what produces the same results, by similar means, is an infringement. What improvement is patentable if this is not? No mere change of proportions of ingredients, if there be no different results would be excused. If such were not the rule, a patent, in no case, would be worth a cent.

So far as identity is concerned, if you are not to guess merely, but rely on the witnesses—if the test of analysis, by competent, scientific men, be of value, then the proof is all on our side. The attempt to quibble and cavil about the language of our specifications is a most ungracious defense; it is a backing out from the ground they originally took. Then they boldly declared we were but pretenders; false claimants to the originality of the invention. Now, the ground is, you may have invented the process; may be deserving of the honor and credit of a valuable application; but you have not described clearly what you have invented. The Defense now seeks to defeat the meritorious work, because the language is not clearly expressed. That faculty indeed is far different from that of invention. There is no faculty of the human mind greater than that; that almost makes a God of man. Those who are the greatest honored with this God-given faculty, are often those least able to describe, least fluent on the great topic of their labors and their thoughts; their mind, drawn within themselves, becomes intensified, loses itself in the common converse with the world. Talk with the best men who invent, they are deficient in this particular. They may tell you of the great thing on their mind, but are at a loss—out of their track. They may have found out the secrets of Nature, but they have not found out the secrets of language. Dr. Allen, it seems, attempted to write his own specifications; they are short.

If a lawyer had written them they would have been twice as long at least.

The law will lean favorably to the specifications ; it deals with men as men. The Judge will construe the language beneficially, like Wills *in extremis*.

They say that in different places the specifications are imperfect, any one of which is competent to destroy the patent. This is not only a question of law, but of professional skill—of law and facts ; it must be testified to by experts. "Swedging," "rimming," "white heat," are terms all new to us ; and upon whom are we to rely ? Experts ; who are they ? It would have been of no additional importance to the public to have lengthened out interminably the specifications ; if folios had been written in explanation *they* would not have known their worth. The language was addressed to those skilled in that art or that allied to it—not to bunglers, but to those who used the compound indicated.

Mr. Mathews says it was addressed to the Dental Profession generally, to the Operative and Surgical Dentists. Not so. The witnesses say that this art comes nearest to block work, and it is of course addressed to those acquainted with that business. They must decide. Nine witnesses (naming them) testify that they took the materials and made the work, from the specifications, without other instruction or "private circular" information. Where are the nine men on the other side, proper persons, I mean block work makers, who say to the contrary ? There are a good many, wbo testify that they can't make the work according to the specifications, but of these, some, I think, can't make it from any formula. Dr. Main did not know what it was even to make a "frit," *he* could not make it from the specifications.

Now as to the particular points, as to defects—they are not defects The law itself says, do not run into too mnch prolixity, let the statements be short, but clear.

We have a right to take it for granted that experts know what is common, and you may take all for granted.

Then as to borax. We have been bored with borax. I have got so tired with the sound that I never want to hear it again. In the argument it was borax, borax, borax, and nothing else. Borax was the Mamelon Tower of their strength—the last hope of the defence seemed to be in borax.

"Take an ounce of borax" that's "crude," they say. Put it into the furnace, the purpose being to fuse the compound on to platina, the least contractible of all metals, borax, every one knows, will expand into a syllabub, it bubbles up like a snow drift. The error we committed, they say, is, that we did not say crude must be used and that the water of crystalization must be drawn off. They say we have not taught these men, but deceived them. What do you make of that ? What do teeth makers tell you ? The Philadelphia man,

Dr. Stockton says, he who puts in borax crude is an ignoramus, if he did not know *that* he does not know his profession. Suppose the operator has not knowledge enough of the properties of borax, would our patent fail?

If at the first experiment he did fail, he would see that would not do. The law says that is ambiguous which requires *repeated* experiments, but the fact is, one experiment in the case is enough.

What other criticism have they? They say, we don't advise the use of platina plate, that this article was almost unknown to Dentists, and when called upon to put the compound on a plate, they would not know certain that a platina plate was meant. Some say they would take platina or palladium. If we had said only platina, they would say, we should also have enumerated palladium, &c.—if one or both had been specially referred to, they would have some cause of equally groundless complaint.

A. in effect told them that silver and gold would not do. Some testify that certain things are refractory and infusible, and certain ones fusible, and that fluxes are used to assist them. Every toothmaker in the U. S. knows that spar, kaolin fluxed and heated, can not be fused on silver and alloyed gold.

He showed specimens of block work, *platina* pins which had been used for twenty years; why were they then and since used? Because they were put in while the mass was plastic, and submitted to a high heat, the same materials in the A. compound show that similar heat would require similar non-contracting metal for a plate as well as for pins. The specification applies to any plate on which you can fuse the compound.

Again, they say we do not advise the best way of doing this work, which is by back straps, for solidity and as stays they are valuable, but work can be with or without them. We say then it was not necessary to say more than we did say; back straps *may* be used. Does that vitiate our policy? Do we conceal the process? No, we offer them their choice, but our opinion is expressed that we can do without them—they have been successfully used without them; whenever they can be on account of their weight, being heavier, they ought to be made without them; the object being to attain strength and lightness, to be lifted by the muscles of the mouth, it is important that the work be light. If they can be dispensed with they should be. The experiments showed reasonably it could succeed without.

This is an adjunct, and not the thing itself, it was then believed to be sufficient. Before the Dental College the inventor had flowed the compound on a plate two inches long, had turned up one end and put an incisor tooth over that without backstraps—it was tried before the class, and stood like "Fides and Achates;" the tooth finally snaps off, but not the attachment. That experiment astonished Dr. Hunter. He almost insinuates fraud. What kind of teeth, he says porcelain teeth. It opened his eyes to what was not dreamed of in his philosophy, it opened his eyes in the direction of what he was aiming at, block work. He said "I will break it with my fingers." His fingers! if it did not burn his fingers, it would break them.

These are the points of our discovery: a silicious cement, an adhering substance, forming an attachment without a cumbrous alliance. As to the complaint of our flux being "any known flux" we give a good one and say besides, "any known flux." The objection on this point is mere hyper-criticism. No man would put up black bottle glass flux, he would use what is usable and what he knew was used.

It is claimed that Hunter's is a better mode of manipulation—the proper mode "make a frit." When I instruct mortar to be made, speaking to a brick-mason, would I say, take sand and take good calcined lime and mix with water? They say fritting is necessary if there be a usual mode, need we say so? Must we describe how to make a frit? It would be a work of supererogation.

As to the blow-pipe; first H. said it was ridiculous to do without it, now he does his work without a blow-pipe.

Then, again, we have concealed an important fact; when the operator is lead to expect one heat is sufficient, more are actually necessary. Our instructions don't bear that inference. Does it require any invention in heating the second time? What is done at the second heat? As a man putties window sash when there is a crack, so does he fill up fissures and re-insert the work in the furnace. Our adversary has tried every rivet of our armor, wherever a thrust could be made, but we stand on a foundation as hard, as endurable as our base, our compound.

Are we the inventors? They say no? Dr. H. presents specimens, Dr. A. presents specimens, and both are as perfeet as human art can make them; but H. says the process is old, aged, long known, thirty years ago at least—if so, why was it not practiced?

As to the "Col. Burr specimen." A specimen of the teeth from the jaws of Aaron Burr! whose hands received them from him? were they taken from his mouth before or after death? Why are they present here? Is it as a sacred relict? Was it worn by Col. Burr when he shot Hamilton, when he loaded his pistol and fired? None can trace this, it is mere rumor. This old, dilapidated specimen is alleged to have been made by Delabarre—they must make another guess. Dr. Jobson, the author, was in Paris in the laboratory of Delabarre, and he says his process was a failure and was abandoned Fitch, his translator, says he tried Delabarre and in doing so, ineffectually spent $1,000—he lost his money and gained no results.

I have no time to go over the miserable attempts made to show that others in the United States made continuous gum work, anterior to Allen; they attempted it, but never succeeded.

Dr. Miller, a witness of great intelligence and of great importance, says the result had never been reached until reached by Allen. A Dentist in New Orleans, says it never was accomplished until it was done by Allen.

Dr. Main brings us a specimen never made before he filed a certain answer—we had filed a bill against him—he then said, "as for me I never made or attempted to make for practical use, any work like yours, none except for philosophical experiment while in my laboratory." This is a different statement from that made on the stand here; quite a difference between New York and Cincinnati. He is no reliable witness, he is mistaken; we cannot lose our case on such testimony.

These were abortive experiments. To attain such desirable results in the dental art, there has been a struggle at home and abroad.

The intellect over the world has been agog on this subject, and complete success realized only in the Great West, at Cincinnati, O.

Are you going to set our invention aside on the ground that some of our ideas were known before, and blot out what Allen has done on account of what Hunter did afterwards? ignore the grandest triumph made in the dental art? go back to the days of such specimens as those presented as being used by Aaron Burr? or to those other specimens whose compound has scaled off, perished, gone?

But let us come to the point on which all this matter turns. Between Allen and Hunter, then, which deserves the credit? what is the thing which determines? A silicious compound, fusing, &c., connecting and adhering to the plate in the strongest manner. Who invented that compound, Hunter or Allen? Take all that Hunter contends for; we may admit that in 1848 Hunter did work like ours, still your verdict must be for Allen. If Hunter did do the first practical work, he, Allen, was the first to conceive the idea, and commenced to experiment. The rule is sanctioned by high authority, as to what results in identically the same thing, one reaching it on Monday the other on Tuesday, the credit and patent is to be given to the man who commenced first. It is not for the perfect thing the patent is given, but for the germ, for the first getting on the road. The law provides for priority of conception, this will settle the case if H. even be nominally ahead in the work.

"It is not sufficient to defeat a patent, already issued, that another person has conceived the possibility of effecting what the patentee has actually accomplished. To constitute a prior invention, the party alleged to have made it, must have proceeded so far as to have entitled himself to a patent, in case he had made an application; or in other words, he must have reduced his idea to practice, and embodied it in some distinct form.* It is true, that in a race of diligence between two independent inventors, our law provides for the priority of conception, by allowing the one who first invents to obtain the patent, if he was using reasonable diligence in adapting and perfecting his invention, although a second inventor has, in fact, first perfected the invention, and reduced it to practice.† But where a patent has been granted to a patentee, who did not surreptitiously obtain his knowledge from a prior inventor, who was using reasonable diligence to perfect and adapt the invention, in order to defeat it on the ground that the patentee was not the first inventor, some previous inventor must not only have had the idea, but must also have carried the idea into practical operation; for he is entitled to a patent, who, being an original inventor, has first perfected and adapted the invention to actual use."‡—*Curtis pp.* 37 *Sec.* 43.

Take him on his own ground, we sue not for what was done before the patent, but for what was done since.

"As to the time of the acts complained of as amounting to an infringement, it is obvious that the patent cannot be infringed by any thing done when the patent did not exist; and therefore it is no infringement to make or use a machine subsequently patented, or otherwise to practice the invention which is afterwards made the subject of a patent, before the patent is obtained. But when a patent is granted, the right of the subject-matter relates back to the time of the invention, so that the party who has practiced the invention between the time of the discovery and the issuing of the patent, must cease to do so. Any acts of infringement done after the issuing of the patent will be ground for the recovery of damages, although the previous acts were done at a tims when it was uncertain whether there would be any patent issued.* The same is true of acts done in violation of a patent which is surrendered and renewed on account of defects in the specification. If a party erect and put in use a patented machene during the existence of a defective patent which is afterwards surrendered, it will be an infringement of the new and renewed patent, if he continues the use of such machine after the renewal: and it seems that no notice of the renewal is necessary; and if it is, that knowledge of the original patent will be notice of the renewed patent granted in continuation of it, according to the provisions and principles of law."†*Ibid pp.* 295–6 *Sec.* 255.

These points however will be given you by the Court.

These assumptions are admitting even that he made perfect work. As early as 1810, Allen conceived the idea, slowly and surely he progressed experimenting; a quick intellect, years afterwards, came in and reached the result, but if Hunter had not been born, Allen would have perfected it. I don't know where they can land. Hunter came here in 1843; none certainly take his conception of the design to have been previous to '46 or '47. He may have heard of what we were after, for he lived in the same city; but having learned our object and pushed his experiments boldly, would it be right for him to rob us? But, gentlemen, he never got on to our track, he did not know what we were at.

I am glad Dr. Hunter is here to hear me, and that these Dentists are here giving their attention to this case. My friends, none are more indebted to these men of art, than myself, to them I am indebted, in an important sense, for my ability to address you today. Hunter was not after the idea which animated us, his ingenuity and ardor was directing in a different route altogether from ours. When he found ours, he ridiculed it; when he found ours would supplant his, he tried to break us down.

There is much in Hunter that I like; he has talent, perseverance, energy. I wish it had been exerted in a better cause. None knew better than Hunter that it was not a silicious compound, but a baked body, H. was after; it was for block-work, not to be fused to, but to be set upon the plate.

Those specimens of Dr. Taliaferro, Mrs. Guilford, the World's Fair specimens, show well, they look like ours, the compound may be fused on the plate; there appears to be a union, but I do'nt know if any of them are united. I do'nt know what lies under the gum; I do not know if the body of those of Guilford and Taliaferro is of the silicious cement, and if so, that he made it, until after he saw our specification. Did you examine the union ? Dr. T. says his was put in in 1848. The persons wearing the jobs don't know. One person who had long worn a set of teeth, had been so non-observant, that when asked if his teeth had back plates, could not answer until he took them out of the mouth, to examine before he answered.

But all these specimens have been in the hands of Hunter, after he saw our work, when we had taught him how to do such work. What he was after, as proved by his own statement, was not what has been exhibited here as work done by Hunter in 1848. No Cincinnati Dentist in that year knew that such work had been done. If it had been done, Hunter would have told of his success to every friend; he is communicative, but he never told it then.

He says, "in 1849 I learned of Dr. W. very valuable information, &c. ;" but what do you want with a new process, you have already got the thing ? If Hunter says he put this work on them, it would take many witnesses to make me believe it. If, then, I make Hunter a witness for Allen, what need is there of a better ? If I find deception in one instance, I must look to find them in another. He brings here the work said to have been made for exhibition at the World's Fair; what the gum is and what the connection really is, we can't tell, (I will break them and show you—said *Hunter.*)

At least this specimen is on silver galvanized. Can our compound be fused on that plate ? (Mr. Matthews said, it can be on platina, and silver soldered over it, as this specimen actually is.) The specimen sent to the World's Fair is not described by McCurdy as it is by Kendall. He here read McCurdy's description. They have presented certain specimens, the case that contained them, the award of the Commissioners; but there is one thing that they did not present, and that was the identical description of his invention written by Dr. Hunter, and sent to the World's Fair.

He has brought all but his own description made at the time. Why should he have sent a gold and silver specimen, to show his method, if the method only can be done on platina? McCurdy, who superintended its exhibition says, it was only block work, and attached to the plate only by wax—there was no metal, no gum attachment. This very gentleman was partner in the house to which he says, he sold his receipt. These men were block workmen ; heretofore they had made blocks in sections, they wanted full arches to sell to Dentists, for them to set on plates. It is astonishing if the story told by Hunter and his witnesses be true, that McCurdy knew nothing about it. I bring the man Hunter as a witness himself. Dr. McC. gives an exact description, but not so exact as Dr. Hunter. Would Dr. H. act intelligently ? would he improve backwards ? would he retrogade? will he forget what he did in 1848? that would be most remarkable. He would send his best article to the World's Fair ; but as McCurdy describes it, it was not equal to what Hunter alleges he made in 1848.

I have admitted, for argument's sake, that Hunter made practical work before we did, but that is not the fact.

We commenced our experiments in 1840, three years before Hunter came here, not to make block work, but a cement that would fuse on to a plate. As early as 1843, Bœhinger saw a specimen of ours, it was a cement fused on a plate—the principle we sought was there, though it was not practical work.

He was on the track but the work was not perfected. In 1843, Penniman, one of the witnesses, saw work, the plate was white metal, not yet practical work; but this was attained in 1847, in the set made for Mrs. Aldrich, (H.'s practical work for Dr. Taliaferro was not made until 1848.) In the year '47, A. made a set also for Mrs. Bartlett, a lady of clear intellect, who testified so clearly before you ; her's were set on a platina plate which was galvanized. We are then first in conception and first in the practical use of our method. Grant then that Hunter made work in 1848, it must be conceded that we made it in 1847.

There remains for me that part of my duty which is most unpleasant, to vindicate my client as a student and an experimenter, who never did or said aught against any body. When he had succeeded, in steps Dr. Hunter. H. has assailed in bitter and unprovoked terms this unoffending man; there is a motive for this, it is an interested one. He saw, instantaneously, that his block work was gone, that a full arch was made and attached to the plate too; here's a man who beats me, who fuses on to the plate, I require solder.

Mr. Matthews places his client on high and disinterested ground ; that his object was not to make money; has Mr. Matthews forgotten the important testimony, that H. sold his secret on pledge of constancy.—The root of all evil sprung up there.

Hunter had been engaged laboriously and usefully for block work; but when he saw our work, and that we were about to make a fortune, were about to pluck the golden fruit, Hunter stepped in to circumvent us. Here's the man (pointing to Hunter) that did this and more. Instantly he set upon our track, he opens upon us in Sept., 1851. He attacked Dr. A., who had never provoked him, publically in a Dental publication, denouncing the boasted improvement as no novelty. That he was a pretender, a copyist of Delabarre, Levett and others; a mixer of glass and sand. He opened his batteries at all points, that he had got an improvement himself; then he said that attempting to stick on the plate was absolutely ridiculous. Now he sticks all on plate. Dr. H. writes us down, before "Steemer" appears, as a Levett Enameler. Levett himself since says his enamel is thin, to flow over bodies not to unite them, and that his enamel differs from our compound. We leave Levett to him. But the worst feature is, Dr. Hunter used Steemer against us. The article of October 17th, 1851, in the Cincinnati Daily Gazette. was written by a Dentist, either by Dr. Hunter himself, or by George W. Kendall. K. paid for its insertion as an advertisement; and this K. was the bosom friend and partner of Dr. Hunter; it was an abusive slanderous article; it charged my client with fraud, with perjury. Before a third person was used, they ought to have been advised that he could be relied upon, that he had tenacity in falsehood, that he would stick; such a man they got. A. did not see Steemer until May, '51; and in April preceding, A. communicates to Dr. Taylor, of the Dental Register, the announcement of his discovery, and that was in practical use, worn in this city. But Steemer recanted; but still Hunter proclaimed Steemer to be the inventor. Somebody had been setting Steemer on, some one was trying to rob him from interested motives, his plans interfered with some one. Until '51 Hunter never dreamed of this process—when seen, he calls it a sticking of gum color.

In that Book, which contains more Truth than any other, it is said, that if you have an enemy you fear, you should pray, O! that my enemy may write a book.

He tried to write us down at the time. My client hoped that he had not an enemy; the blows of his assailant he received calmly, hoping that in time truth would come. He has been challenged into this very court, and the wrong doing of H. he may cover up awhile but he cannot eradicate the evidence.

His card, as McCurdy calls it, he does not produce; his own description of the specimens he sent to the World's Fair. We recited to you the description given by Mr. McCurdy.—See Dental Recorder, —— No., 1851, for article signed by Wm. M. Hunter himself.—"Steemer" is his key note, first and last, he never lets go of Steemer, even after Steemer let go of us. Hunter describes his own new and important plan as improved block work, arched block work; in his own language, "to make block work without carving, &c." He no doubt arrived at an original result of block work, but he has no such phrases as "fusible cement, to fuse, to plate," it was "block work," and that was all.

He refers to McCurdy for the truth of his description; he has a plate, with a silver plate afterwards united; but that is not our improvement, that was what we tried to avoid, that's not Allen's improvement, that's Hunter's "block work, &c."

Going back to Sept., '51,—before Steemer's article,—he heard we had made some improvement—he sent for our specification, but had not received it. We had not written against him. He volunteers then, in behalf of the profession, to protect them, not to secure his own interest. Sept., '51, Dental Recorder, he writes to forestal us, to break us down; he says of our work, it is not worth anything, not to be relied on, fit only for show, a humbug; this was before he got our specification. Then he come out Dec., 1851, "new method of construction of artificial teeth," of a "body" not materially contracting in the fire; he not knowing what ours was, said, that he would break it between his fingers, that it was enamel alone; is ours an enamel? He said there was no strength in platina; he condemns platina, and ridicules the silicious cement. Let him break our teeth, and Moses Kendall, our operator, will mend them in three hours. All this time there is no word, no hint of having put specimens in the mouth in 1848.

After he got our patent specifications, by despatching specially to Washington for them, he comes out with a new publication, it bears date Nov., 1852; it bears the title of a "new method," with the apt quotation "can there be anything new?" For his granulated body he is indebted to Audebrand. This man Hunter has philosophy, has science, he is near to us, but not quite up to us; he has got only an imitation of ours; he injures his compound by his granulated body. But they say that Allen, in 1849, bought of Wildman a receipt which opened to him a new field. He would lead you to believe that his eyes were only opened in 1849—when he had made work that adhered so, that the fingers of Hercules even could not break it.

H.'s compound is grains and powder; our's is subject occasionally to crack, his may not; but his does not as successfully adhere to the plate. The contact of a grain at one point, is like that of a ball on the plate; but if flattened like ours, it has a large connecting surface.

I am about done with this case; I am reminded by my client of the defiance and denunciation shown and hurled at Dr. A. by this defendant. He has termed him a pretender, a liar, and has for years pursued this unoffending man, without any provocation, with bitterness, aye, too much bitterness; but he has waited patiently, and at last accepted his challenge to bring the difficulty to a suit.

He was bound to sue; Hunter and Kendall had libelled him, publicly laughed and jeered at him, and insulted him by declaring that "he would give his head for a foot-ball if he did not put us down." He has not put us down. My client has pursued his labors, abused no one. The conspirators had followed him with a vindictive spirit. It is for you to say now, if this Defendant shall triumph, if you will send my client out a ruined man. Deliberate well! Take care, and give a verdict that you will afterwards be satisfied with. Give the Plaintiff his rights; we do not want the money of the Defendant. We want a verdict, and that we are entitled to at your hands.

Mr. Stanbery closed his argument at 4 P. M., Saturday.

CHARGE OF THE HON. JUDGE JOHN McLEAN.

*Gentlemen of the Jury:*—This case has been put on trial before you, that its merits may be calmly and deliberately investigated without reference to previous excitement.

The Patentee claims to have invented a new and useful mode of setting mineral teeth, etc., (he here quoted the language used in describing the compound, the gum color, and its mode of application).

By saying this is the compound which he "prefers" he does not mean to say it is the only compound which will make it: for his language is "any known flux."

After showing how the application is made, the Patentee says: "I claim a patent for 'a new mode,'" etc. This is the summing up, expressed in a few words, of what he has invented.

In order to secure what he considered a valuable discovery he entered in the Patent Office his "Caveat" on the 29th April, 1851.

A Caveat is entered to preserve the right of the discoverer and to enable him to perfect his invention. It is a notice to the world of that fact, and time is given him so to perfect it, during which the law protects him against an application for a patent for the same by others. The discoverer and inventor, on the 23d day of December of the same, obtained from the proper United States authorities a patent for the "new and useful mode" in controversy.

A good deal has been said as to the policy of physicians obtaining patents for discoveries and inventions immediately promotive of the health and happiness of patients, or the elevation of the character and means of usefulness of the profession. We who are the triers of this controversy are not to enter into the consideration of any policy, we are governed by law. The law has thought proper to secure rights to any one, which he is entitled to by discovery or invention, the monopoly is thereby taken from the public and given to the individual. The discoverer only receives protection for ten years to compensate him for the labor expended, and it is never renewed unless it is shown that the patentee has not been compensated for his genius and labor by the first patent. The whole policy of the law is to provide for those who are deserving. Look abroad, and it will be seen that the most useful means in life are aided by inventors, and it is a notorious fact that inventors are poorly rewarded.

But from the feeling engendered in the professional contest we are to free ourselves, and divest our minds of its influence.

The grounds set up by the Defence are various.

1. It claims that the patent is void, on its face, from ambiguity; for the want of certainty in the specifications.

The law requires that an inventor or discoverer shall swear that he veritably believes he is the true original discoverer or inventor, as the case may be, and he must make a clear and specific written statement or description of what he solicits to be patented, so as to exactly distinguish it from other inventions or discoveries, that the public may be enabled to make the compound and use the same.

The Patentee, it is contended, has taken ground too broad, and therefore his patent is void, as he, in describing the "cement," says, it may be formed by the use of "any known flux," thus exacting that experiments be made, and causing the operator to use one or more fluxes which may be found not to suit.

The words of the specification are not to be dissevered; they are to be taken together, for it is the policy, in England and in this country, to sustain a patent where it substantially gives the notice that the law requires. It is insisted that all "known fluxes" is too broad a term to use, and that if there are known fluxes that *can't* be so used, it voids the patent; that the language is addressed to ordinary dentists, to those of medium ability, not to the most learned, that those who are to practice the dental art are those to judge. Who does the law make the judge of this part of the specification. The law says it must be so certain that a person skilled, etc.

The compound in question is a chemical one. Go then to the men who understand chemistry; not to the most ignorant, not to the most learned. They are the experts to be relied upon. Now, gentlemen, would you refer this matter to the mechanic, to the carpenter, to the shoemaker, either of whom may know as much of chemistry as dentists generally?

As to borax, whether the crude, calcined, or fritted, there is said to be an uncertainty in the recipe given for the use of this article. The same is remarked as to platina, a metal which it seems is the only one on which the compound can be fused. These doubts are not only addressed to the Court, but beget mixed questions of law and fact, for qualified experts to resolve, for the jury finally to determine the sufficiency of these specifications of the Plaintiff. The making of this disputed compound is not necessarily connected with the business of the dentist. A small portion of them attempt to make the article themselves; the greater number prefer to purchase as they do the teeth, already made.

The law requires the specification to be so full and clear, so certain as to enable a person to make the compound—a person who understands the compound and the ingredients of which it is composed,—you have no light to guide you except that furnished you by those who are properly experts.

Dr. Clark is one of the first experts called; he testifies he could make it from the specification. Dr. Hazlett bears the same testimony. Van Emon says he can make it. So says Dr. Chapman. Dr. Robinson says the work is superior. Dr. Staughton says a Dentist of proper information can do the work. Dr. Barlow says it requires much skill, but he made it. Dr. Kingsley, a manufacturer of teeth, says he would know that borax must be "fritted." Dr. Semple bought the compound with special instruction, at a loss how to use the borax without it. Dr. Wardle says a manufacturer of ordinary skill could make it. One testifies that he put the compound in fire three times; once he failed. Dr. Babcock tried, but could not make practical work. So Porter. Dr. Smith tried and abandoned it. One could not set the teeth without back plates. Doughty and Colman tried it without success. Dr. Main says he boxed the compass in trying to make A. prove practically successful. Dr. Hays says A. cannot be made without experiment.

These and other gentlemen have been called as experts.

In coming to your conclusion on this point gentlemen, you will be governed by the knowledge that the witnesses seemed to possess, for without that their opinion is valueless. Enquire as to their competency to speak of this matter.

The next question gentlemen, will be as to the infringement charged against the Defendant—that is, using the thing patented. No colorable alteration will except one from responsibility. If a person claims immunity under an attempt at improvement, he can't use it, unless he applies for a patent as an improvement. It must be substantially different, not colorably, slightly different, it must be different in principle; substantial means must be used to accomplish the result, a mechanical contrivance by which a result is produced, a slight change will not do. The law protects the substantial results of the Patentee. On this head a certain publication in the Dental Recorder and Register was read, showing the formula of the defendant, which the plaintiff alleges is the same as his own.

Dr. Slack, a chemist of established character, who has analyzed the formulas of both, says, that Hunter's is substantially and as effectively the same as Allen's, as that 2 and 2 make 4, or that 3 and 1 make 4. This result is shown by analyzing the ingredients of which the compound is formed.

J. M. Locke, Jr., has had analyzed under his direction, the formula of Hunter, printed in Sept., 1852, and that of Dr. Allen, and testifies clearly that there is no substantial difference between them—this says a Mr. Kendall, and so says other witnesses.

The Defence has introduced able persons, skilled in the art, who testify that they consider the formulas widely different—one is fine, the other a granulated body.

It is for you to determine and consider the weight of the testimony on each side, and come to a proper conclusion under all the surrounding circumstances of the case. Next, the discovery or invention must be useful; whether the credit of originating the one in controversy rightfully belongs to the Defendant or Plaintiff; the concurrent testimony of most of the witnesses is that it is exemplarily useful. Dr. Jobson, of London, from his appearance on the stand, a very intelligent gentleman, said that it was the most remarkable improvement of the day, he corroborates others as to its usefulness.

It has been well remarked in the discussion, than in an invention or discovery the result or mode should not only be better than any other, but that it should be useful. So far as this one is concerned, if carried out by the plan of the patent, and of that you are to be the judges, it is understood to have accomplished the most valuable purpose.

The important inquiry is, whether this pantentee is the first and original inventor; he must unite both; he must be not only the first but the original one; if any one made the discovery before he did, although it was not patented, it is fatal to the patent; he must sustain not only its originality but its priority. A man may make a discovery of what had previously been discovered; if such a discovery has previously been made, it is fatal. It is claimed that Fitch's published translation of the French author Delabarre, contains the formula from which the plaintiff must have obtained the substance of the one for which he has Letter's Patent.

If this work contains a formula substantially the same as that of the Patentee, the patent is void, and to this matter experts have been examined, and on that it is to be determined. Dr. Clark very plainly says that Delabarre's formula was somewhat like this; but having been found impracticable, was abandoned. Dr. Jobson was acquainted with D. in France, and he and eminent Dentists had agreed in their testimony, that his plan had not been successful. Whether abandoned or not, if Delabarre's plan contained the materials of which Dr. Allen's formula is composed, it is fatal to the patent.

Several others speak of the process having been abandoned in this country.

The Defence produce teeth, said to have been worn by Aaron Burr and also, perhaps truly, said to have been made by Delabarre himself, you can have that specimen with you gentlemen to examine and compare with those produced by the plaintiff. Some of the witnesses consider this work to be similar or very like that of

Dr. A. It must be substantially alike to defeat the patent. Dr. Fitch testifies that Delabarre never, to his knowledge, brought the work to practical use. The defence also maintain that the Steemer formula contains the substratum of the Allen one. What he is reported to have said in either of his cards or in his testimony in this case, you the jury must weigh, to test its worth and influence in this case, and his credibility opposed to other witnesses.

Dr. Laing testifies that in 1835 he was acquainted with the plan of work executed by Dr. Dodge. One witness says he met with such work, a specimen which appeared to have been in practical use, and gum work was brought to his office for repair.

The Defendant himself claims to have discovered the same thing before the Plaintiff, if so the Plaintiff's patent is inoperative and void.

Mr. Aldrich states that Allen in the fall of 1847 made a set of teeth for his wife, the witness being the brother-in-law of Dr. Allen. The sister of Dr. Allen testifies that she had one set made by him, which appeared outside and in to be of continuous gum, these were exhibited to you. (Judge McLean narrated cursorily the important testimony on both sides which it would be desirable to recapitulate in the light presented by the Judge, if space would permit.)

This is the evidence given or read in your hearing gentlemen, covering the principal points of the controversy which you are to pass upon.

It seems that both gentlemen have been experimenting for some time, and the question has been presented to me to give you the law as to the first inventor.

It is not sufficient to defeat a patent if a person has conceived the possibility of effecting what the patentee has accomplished. The party alleged to have made the work must have proceeded so far as to be entitled to a patent. You are to determine who has the priority of invention claimed by the Plaintiff.

It is alleged by the Defendant and in evidence that Dr. Hunter, in fall of 1850, sold to Dr. White of Philadelphia a formula, published in 1852, which is said to be similar to that of Plaintiff.

This very important subject, gentlemen, is one of no ordinary character, and no ordinary feeling has characterised the case up to this point, but we are not to decide from passion, we are to decide the case entirely on its merits, and I trust you may come to a satisfactory conclusion. The Plaintiff prosecutes not for damages but to establish the patent, if the Defendant be found to have infringed, you need give only nominal damages, say six cents; if you find he has not infringed, your verdict will be "Not Guilty."

This whole subject so deeply interesting to the parties and not so uninteresting to the public is now committed to your determination.

At 5 P. M. the Judge concluded his charge and the Jury retired.

At 5 30 P. M. the Jury returned with a verdict of "*Not Guilty*,"

which was ordered to be recorded, and thereupon Mr. Stanbery on the part of the Plaintiff filed the following motion:

JOHN ALLEN,
*vs.*
W. M. HUNTER.

The Plaintiff moves the Court for a new trial in this case on the following grounds:

1. That the verdict is contrary to the evidence.
2. That it is contrary to law.

STANBERY, NEWTON & MINER *for Plaintiff.*

MEM.—The Reporter would have been glad to have submitted his proofs to Judge McLean, Mr. Stanbery and Mr. Newton if they had been in the city while it was being prepared for the press.

---

### *Great Dental Patent Case.*

(Copyright Secured.)

The first form of the Report, as published, was printed before we had revised the proof, and it will be found that the name of Delabarre, although properly spelled—yet the letters are not rightly arranged.

On page 275, second line from the top, one "e" should be omitted, in "expertness." ED.

---

A CARD.—The Dental Profession have already become aware, no doubt, of the result of the suit between Dr. John Allen and myself, recently tried in the Circuit Court of the United States for the Southern District of Ohio. Lest they may be injuriously misled, with reference to the decision, I desire to make this statement. The validity of the patent remains untouched by the verdict, which was a general verdict of "not guilty" of the infringement charged. The only questions involved in the issue, *decided by this verdict*, are those which are raised with reference to the sufficiency of specifications attached to the patent, and the alleged infringement by the Defendant. The verdict of the jury must be based on the belief, either that the specifications were insufficient, or that there was not sufficient proof of infringement. The patent remains, as to the question of its validity, as it did before.

I desire, also, to state that the unpleasant controversy between Dr. Allen and myself, which led to the suit, has been amicably reconciled since its termination; and that while I announce that my own private and professional engagements make it impossible for me to engage in teaching the benefits to be derived from my system of manipulations in the employment of a dental substitute, John Allen is fully authorized to combine with his own system, under his patent, any advantages he or the public may find in any ideas that have been regarded as peculiarly my own. I therefore cheerfully retire from all controversy and competition with him, and recommend him to the profession, for those *oral and practical instructions* in continuous gum-work, without which few can practice it successfully and advantageously. The compensation which he charges is small, compared to the value of his instructions, and will be a much cheaper and safer method of obtaining the right to pursue the practice and the information and skill necessary to pursue it profitably, than the certain expense and trouble and uncertain result of defying his right as patentee. WM. M. HUNTER.

"*Quantum suff., nuf sed.*"

Cincinnati, June 4, 1755.

P. S.—Since writing the above Card, in the Recorder, I have been exceedingly amused at the reasons volunteered by those of a *certain class* as to the cause of the publication of it.

I choose only to say, at present, to those who would have gloried in my defeat, and who draw upon their imagination for a cause of its publication, that I am in no way indebted to them for my success; and whilst I have a right to do as I please, they should expect nothing at my hand. As ye have made your bed so may ye *lie* upon it.

"Let Hercules himself do what he may,
The cat will mew, and dog will have his day."

W. M. H.

---

Card.—The subscriber invites attention to the following statement, with reference to his improved method of setting Artificial Teeth with continuous gums, in connection with the late trial in the United States Circuit Court for the Southern District of Ohio, in the case of Allen *vs.* Hunter.

The Defendant pleaded the general issue, which involved the question of priority, sufficiency of specifications, utility, etc., etc. As to the question of priority, it was urged on the part of the Defense, that Delabarre, of France, has produced the same results as the patentee, some thirty years ago.

Testimony of the highest order, from Europe and America, was introduced by the Plaintiff, showing that Delabarre had never perfected his plan, or brought it into practical use.

Chemists of the first rank in America testified that they had analyzed Delabarre's formula, and found, by the ultimate analysis of the ingredients, and by actual experiments, that the nature and proportions of the materials were such that good practical work could not be produced, therefore Delabarre's experiments, or those of any other, proved no bar to the patent.

As to the sufficiency of the specifications, although some six days of trial were spent upon them, and the Judge charged the jury specially on this point, that, if they found them defective or wrongfully obtained, they should *void the patent,* still the patent stood the test unscathed.

There were two points of issue. The most important was to test the validity of the patent, which was sustained by the verdict.

The other was that of infringement, which was lost in consequence of the Plaintiff not being sufficiently fortified on that point.

He relied on testimony which went to show that Defendant had sold formulas and given instructions for producing the same practical results, but there seemed to be no law against selling formulas or instructing others, therefore that kind of testimony had no weight with the jury, and the Plaintiff had produced in evidence but one piece of work done by Defendant prior to the commencement of the suit, and that was gratuitous, and the jury returned a verdict of not guilty of infringement.

The testimony of Drs. Slack and Locke, (Chemists, of Cincinnati), showed that although there was an apparent difference in the two formulas of Allen's and Hunter's, yet the *ultimate analysis* of both proved them to be one and the same thing, while Delabarre's was entirely different, and requires thirty-seven hundred and sixty-one degrees of heat to fuse it, while Allen's and Hunter's required twenty-one hundred and four.

Delabarre's formula was published some thirty years ago; Allen's, December 1851; and Hunter's in September, 1852. The two latter differed somewhat in manipulation, yet producing substantially the same practical results.

Since the close of the trial an amicable adjustment of all differences between the parties has been made, by which all further controversy in this case is at an end. The patentee will, hereafter, exercise the entire and exclusive control of this improvement, and will do all in his power to diffuse it among those who desire it.

And he would further state, that he is deputed by Dr. Hunter to impart any ideas in manipulations peculiarly his own in this style of work.

The practicability and utility of this method of Setting Artificial Teeth, has now become so well tested that there is no longer any doubt of its great superiority over all other methods now known. Some of the witnesses in this case testified that they had inserted fifty sets, some ninety, some between two and three hundred, and others between four and five hundred sets, and consider this method superior to all others.

Those desiring an interest in this style of work, will please address,

J. ALLEN,
30 *Bond Street, New York.*

